# Survival Advantage of Peritoneal Dialysis Relative to Hemodialysis in the Early Period of Incident Dialysis Patients: A Nationwide Prospective Propensity-Matched Study in Korea

**DOI:** 10.1371/journal.pone.0084257

**Published:** 2013-12-30

**Authors:** Ji-Young Choi, Hye Min Jang, Jongha Park, Yon Su Kim, Shin-Wook Kang, Chul Woo Yang, Nam-Ho Kim, Jang-Hee Cho, Sun-Hee Park, Chan-Duck Kim, Yong-Lim Kim, Clinical Research Center for End Stage Renal Disease (CRC for ESRD) Investigators

**Affiliations:** 1 Department of Internal Medicine, Kyungpook National University School of Medicine, Daegu, Korea; 2 Department of Statistics, Kyungpook National University, Daegu, Korea; 3 Division of Nephrology, Ulsan University Hospital, University of Ulsan College of Medicine, Ulsan, South Korea; 4 Department of Internal Medicine, Seoul National University College of Medicine, Seoul, Korea; 5 Department of Internal Medicine, Yonsei University College of Medicine, Seoul, Korea; 6 Department of Internal Medicine, The Catholic University of Korea College of Medicine, Seoul, Korea; 7 Department of Internal Medicine, Chonnam National University Medical School, Gwangju, Korea; 8 Clinical Research Center for End Stage Renal Disease in Korea, Daegu, Korea; University of Leicester, United Kingdom

## Abstract

**Background:**

The impact of dialysis modality on survival is still somewhat controversial. Given possible differences in patients’ characteristics and the cause and rate of death in different countries, the issue needs to be evaluated in Korean cohorts.

**Methods:**

A nationwide prospective observational cohort study (NCT00931970) was performed to compare survival between peritoneal dialysis (PD) and hemodialysis (HD). A total of 1,060 end-stage renal disease patients in Korea who began dialysis between September 1, 2008 and June 30, 2011 were followed through December 31, 2011.

**Results:**

The patients (PD, 30.6%; HD, 69.4%) were followed up for 16.3±7.9 months. PD patients were significantly younger, less likely to be diabetic, with lower body mass index, and larger urinary volume than HD patients. Infection was the most common cause of death. Multivariate Cox regression with the entire cohort revealed that PD tended to be associated with a lower risk of death compared to HD [hazard ratio (HR) 0.63, 95% confidence interval (CI) 0.36–1.08]. In propensity score matched pairs (n = 278 in each modality), cumulative survival probabilities for PD and HD patients were 96.9% and 94.1% at 12 months (*P* = 0.152) and 94.3% and 87.6% at 24 months (*P* = 0.022), respectively. Patients on PD had a 51% lower risk of death compared to those on HD (HR 0.49, 95% CI 0.25–0.97).

**Conclusions:**

PD exhibits superior survival to HD in the early period of dialysis, even after adjusting for differences in the patients’ characteristics between the two modalities. Notably, the most common cause of death was infection in this Korean cohort.

## Introduction

The incidence and prevalence of end-stage renal disease (ESRD) has increased globally over the past 30 years [1], [2]. The trend is similar in Far East Asian countries. In Korea, the incidence and prevalence of ESRD was 181.5 and 1144.4 patients per million population, respectively, at the end of 2010 [3]. Approximately four-fifths of ESRD patients in Korea are treated with either hemodialysis (HD) or peritoneal dialysis (PD) as a maintenance renal replacement therapy. Given the growing population of ESRD patients and its heavy economic burden in the medical care system [3], [4]. the choice of dialysis modality is an important issue.

Even though the use of dialysis is determined by both medical and non-medical factors [5], evaluating whether differences exist in the mortality outcomes of HD and PD is of considerable interest. Cohort studies in the 1990s showed similar survival between two modalities within the first 1 or 2 years after initiating dialysis but a higher risk of death for patients treated with PD after 2 years [6], [7]. Recent studies including more contemporary cohorts have shown similar, or even better, survival in PD compared to HD in the early period, but the survival advantage for PD decreases over time [8], [9], [10], possibly due to recent advances in PD technology and improved outcomes, especially in the early dialysis period [11]. Patient characteristics, relative use of PD vs. HD, and the mortality rate for each modality vary considerably across countries. Thus, a modality comparison in a contemporary Asian population of ESRD patients would be helpful for understanding the difference in PD and HD outcomes.

We performed a comparative study of survival in Korean patients with incident ESRD undergoing PD or HD. The primary objective was to compare all-cause mortality between PD and HD using intention-to-treat analysis in the propensity score matched-pair cohort. The secondary objective was to compare mortality risk in the entire cohort and subsets defined by age, sex, and diabetes status.

## Materials and Methods

### Study Cohort

We conducted a nationwide prospective observational cohort study in Korean patients with ESRD (NCT00931970). Patients who were at least 20 years old and began treatment with maintenance dialysis due to ESRD within 3 months were eligible for the study. Patients scheduled to receive kidney transplantation within 3 months were excluded. From September 1, 2008, to June 30, 2011, a total of 1,413 patients were screened and 1,060 patients enrolled from 31 centers affiliated with the Clinical Research Center for End Stage Renal Disease (CRC for ESRD) (Figure S1). All patients provided written informed consent before inclusion and the Institutional Review Board of each center approved the study protocol. [The Catholic University of Korea, Bucheon St. Mary’s Hospital; The Catholic University of Korea, Incheon St. Mary’s Hospital; The Catholic University of Korea, Seoul St. Mary’s Hospital; The Catholic University of Korea, St. Mary’s Hospital; The Catholic University of Korea, St. Vincent’s Hospital; The Catholic University of Korea, Uijeongbu St. Mary’s Hospital; Cheju Halla General Hospital; Chonbuk National University Hospital; Chonnam National University Hospital; Chung-Ang University Medical Center; Chungbuk National University Hospital; Chungnam National University Hospital; Dong-A University Medical Center; Ehwa Womens University Medical Center; Fatima Hospital, Daegu; Gachon University Gil Medical Center; Inje University Pusan Paik Hospital; Kyungpook National University Hospital; Kwandong University College of Medicine, Myongji Hospital; National Health Insurance Corporation Ilsan Hospital; National Medical Center; Pusan National University Hospital; Samsung Medical Center, Seoul; Seoul Metropolitan Government, Seoul National University, Boramae Medical Center; Seoul National University Hospital; Seoul National University, Bundang Hospital; Yeungnam University Medical Center; Yonsei University, Severance Hospital; Yonsei University, Gangnam Severance Hospital; Ulsan University Hospital; Wonju Christian Hospital (in alphabetical order)]. All clinical investigations were conducted in accordance with the guidelines of the 2008 Declaration of Helsinki.

### Data Collection

Baseline information at enrollment included age, sex, height, weight, primary renal disease, comorbidities, laboratory data, and dialysis information. Comorbidities, laboratory data, and dialysis information were followed at 3 and 6 months after the start of renal replacement therapy and then at 6-month intervals thereafter. Comorbid conditions included a history of congestive heart failure, coronary artery disease, peripheral vascular disease, arrhythmia, cerebrovascular disease, chronic lung disease, peptic ulcer disease, moderate to severe chronic liver disease, connective tissue disease, and malignancy. Laboratory data were available for hemoglobin, serum blood urea nitrogen, creatinine, albumin, calcium, and phosphorus levels. The 24-hr urine volume was also measured. The glomerular filtration rate (GFR) was estimated from the Modification of Diet in Renal Disease (MDRD) equation. Dialysis modality was defined as the modality 90 days after the first dialysis, or the modality at dialysis initiation if death occurred before 90 days. Data were collected using a web-based platform (http://webdb.crc-esrd.or.kr). Date and cause of death were reported within 1 month after the event and ascertained by data from Statistics Korea. Patients were censored at the time of kidney transplantation or December 31, 2011.

### Statistical Analysis

Patient characteristics were compared using the Pearson chi-square test or Fisher's exact test for categorical variables and the Student’s t-test for continuous variables. To balance the baseline characteristics of patients, we estimated a propensity score, which is a predicted probability of PD in all patients who survived at least 90 days after the initiation of dialysis using a logistic regression model. The model was constructed with age, sex, diabetes, 10 cormorbid conditions, body mass index (BMI), hemoglobin, serum blood urea nitrogen, creatinine, albumin, calcium, phosphorus, and 24-hr urine volume. Using the Greedy match algorithm, we created propensity score matched pairs without replacement (1∶1 match). We estimated Kaplan-Meier survival in the matched-pair cohort and compared them using the log-rank test. The Kaplan-Meier method tends to overestimate death probability compared with the competing risks approach. Therefore, we used the cumulative incidence function (CIF) to describe the cause-specific survival, with transplantation during the follow-up being a competing risk event. Gray’s test was also used to examine the HD vs. PD survival difference.

Subsequently, we fit a Cox proportional hazard model to estimate the relative hazard ratio (HR) of mortality for PD compared to HD. After analyses in the matched-pair cohort, we re-examined differences in survival and relative risk between PD and HD in an unmatched whole cohort from day 0 (initiation of dialysis) and day 90 (same as in propensity score matched analysis). All covariates used to estimate the propensity score were treated as covariates in multivariable Cox regression. The assumption of proportional hazards was tested using Schoenfeld residuals within the PHREG procedure of SAS. Sensitivity analyses were performed with subgroups defined by baseline age (<65 years, ≥65 years), sex, and diabetes status. Modality changes were not incorporated into the models because only a few patients (n = 29, 2.7%) experienced modality changes during the follow-up period.

The percentage of missing data was <5% (3.1∼4.3%). We handled missing values by creating a missing indicator for categorical variables and imputing the means or medians of existing values by dialysis modality for continuous variables. No information was missing on age, and sex. All statistical analyses were performed using SAS system for Windows, version 9.2 (SAS Institute Inc., Cary, NC) and R (R Foundation for Statistical Computing, Vienna, Austria; www.r-project.org). A two-sided *P*-value <0.05 was considered significant.

## Results

### Patient Characteristics

The characteristics of patients based on dialysis modality in the entire population and the propensity score matched population are presented in Table 1. In the total cohort, the proportion of patients undergoing HD and PD was 69.4% and 30.6%, respectively. PD patients were younger and had a lower proportion of diabetes as the primary renal disease, lower prevalence of chronic lung disease and malignancy as comorbid conditions, lower BMI, higher hemoglobin level, and larger 24-hr urine volume at the initiation of dialysis than HD patients. Among 1,060 patients, 1,022 patients survived for 90 days after the initiation of dialysis. For propensity score matching, 278 pairs were selected from these 1,022 patients. The estimated distribution of propensity scores was similar after matching (Figure S2), and the patient characteristics did not differ by dialysis modality in the matched population (Table 1). The matched and unmatched populations are compared in Table S1.

**Table 1:** Patient characteristics in all and matched population.

Characteristics	All (n = 1,060)						Propensity-score matched (n = 556)†		
	HD (n = 736)		PD (n = 324)		P value		HD (n = 278)	PD (n = 278)	P value
Age at initiation of dialysis (years)	58.1±14.0		51.1±13.4		<0.001		51.9±14.5	51.6±13.0	0.770
Sex (Male%)	444 (60.3)		194 (59.9)		0.892		169 (60.8)	168 (60.4)	1.000
Body mass index (kg/m^2^)	23.3±3.4		22.7±3.3		0.005		22.7±3.0	22.7±3.3	1.000
Primary renal disease, n (%)									
Diabetes	367 (56.4)		145 (48.2)		0.003		130 (50.8)	126 (47.7)	0.282
Hypertension	119 (18.3)		59 (19.6)				47 (18.4)	53 (20.1)	
Glomerulonephritis	93 (14.3)		71 (23.6)				47 (18.4)	62 (23.5)	
Others	72 (11.1)		26 (8.6)				32 (12.5)	23 (8.7)	
Comorbidity at initiation of dialysis, n (%)									
Congestive heart failure	103 (14.6)		42 (13.2)		0.563		37 (13.3)	36 (12.9)	1.000
Coronary artery disease	98 (13.9)		35 (11.0)		0.228		27 (9.7)	28 (10.1)	1.000
Peripheral vascular disease	69 (9.8)		20 (6.3)		0.073		16 (5.8)	15 (5.4)	1.000
Arrhythmia	17 (2.4)		5 (1.6)		0.489		3 (1.1)	4 (1.4)	1.000
Cerebrovascular disease	88 (14.5)		30 (9.4)		0.170		16 (5.8)	20 (7.2)	0.606
Chronic lung disease	88 (12.5)		16 (5.0)		<0.001		17 (6.1)	15 (5.4)	0.856
Peptic ulcer disease	55 (7.8)		21 (6.6)		0.523		16 (5.8)	21 (7.6)	0.497
Moderate to severe chronic liver disease	17 (2.4)		10 (3.1)		0.529		10 (3.6)	10 (3.6)	1.000
Connective tissue disease	70 (9.9)		33 (10.3)		0.843		24 (8.6)	29 (10.4)	0.564
Malignancy	60 (8.5)		8 (2.5)		<0.001		14 (5.0)	8 (2.9)	0.277
Laboratory data at initiation of dialysis									
Hemoglobin (g/dL)		8.8±1.7		9.1±1.6		0.002	9.2±1.7	9.1±1.6	0.516
Blood urea nitrogen (mg/dL)		80±37		78±36		0.403	78±32	79±36	0.680
Creatinine (mg/dL)		8.2±3.5		8.5±3.6		0.182	8.4±3.3	8.6±3.7	0.717
Albumin (g/dL)		3.3±0.7		3.4±0.7		0.224	3.5±0.7	3.4±0.7	0.324
Calcium (mg/dL)		7.8±1.0		7.8±1.1		0.950	7.8±1.1	7.7±1.1	0.790
Phosphorus (mg/dL)		5.4±1.9		5.5±1.8		0.753	5.5±1.7	5.5±1.8	0.705
Estimated GFR (ml/min/1.73 m^2^)		7.5±4.4		7.3±3.6		0.354	7.1±3.2	7.2±3.6	0.630
Urine volume (ml/day)		637±615		768±674		0.002	783±674	764±665	0.740

Propensity-score matching was done in patients who survived until day 90. Data are expressed as number (%) or mean±standard deviation. P-values were estimated by chi-square, Fisher’s exact and student *t* tests as appropriate. Abbreviations: HD, hemodialysis; PD, peritoneal dialysis; GFR, glomerular filtration rate.

Although some baseline characteristics were different between HD and PD, the percentage of censoring due to kidney transplantation was similar between HD and PD (n = 21, 2.9% vs. n = 14, 4.3%). Twenty-nine patients experienced a modality change during the follow-up period (19 PD to HD; 10 HD to PD). HD patients used vascular access at day 90 as follows: native (n = 165, 22.4%), synthetic graft (n = 44, 6.0%), tunneled catheter (n = 302, 41.0%), and non-tunneled (dual lumen) catheter (n = 134, 18.2%).

### Comparison of Survival from Day 90 in Propensity-matched Population

During the mean follow-up of 16.3±7.9 months, a total of 113 (10.7%) all-cause deaths were reported. The crude mortality rate was 78.5 per 1,000 patient-years [95% confidence interval (CI) 64.0–92.9]. Nineteen deaths (16.8%) occurred within 90 days of beginning dialysis (Table S2). The distribution of death events within 90 days was not different between HD and PD. Infection was the most common cause of death in both modality groups (PD 41.7% and HD 29.2%; Table 2). Of the 113 deaths overall, 36 (31.9%) was from infection [26 HD (29.2%) and 10 PD (41.7%)]. The causes of death in the 26 HD patients included pneumonia (n = 12), sepsis (n = 10), intra-abdominal infection (n = 3), and infective endocarditis (n = 1). Of the 10 PD patients, 4 had peritonitis, 2 had pneumonia, and the other 4 had sepsis other than PD peritonitis. PD peritonitis accounted for 4 (16.7%) of the overall PD deaths (n = 24).

**Table 2:** Causes of death by dialysis modality (n = 1,060).

Cause of death	HD	PD	Total
Cardiovascular disease	13 (14.6%)	2 (8.3%)	15 (13.3%)
Infectious disease	26 (29.2%)	10 (41.7%)	36 (31.9%)
Cerebrovascular disease	3 (3.4%)	1 (4.2%)	4 (3.5%)
Sudden death	8 (9.0%)	3 (12.5%)	11 (9.7%)
Cancer	16 (18.0%)	2 (8.3%)	18 (15.9%)
Other*	12 (13.5%)	2 (8.3%)	14 (12.4%)
Unknown	11 (12.4%)	4 (16.7%)	15 (13.3%)
Total	89 (100.0%)	24 (100.0%)	113 (100.0%)

Others included liver disease, gastro-intestinal disease, endocrine or hematologic disease, chronic obstructive lung disease, and suicide etc.

**Figure 1:**
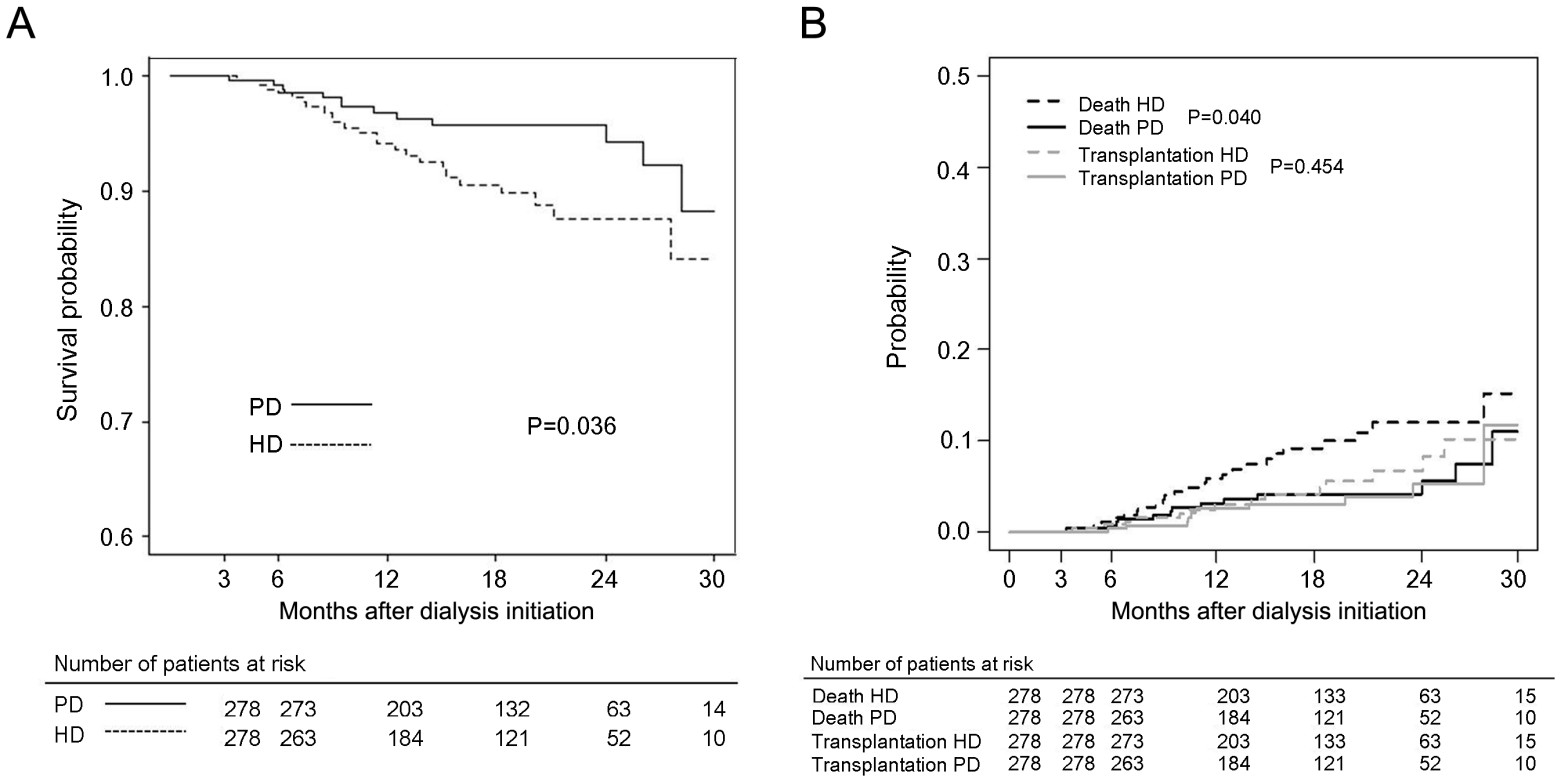
Survival probability from day 90 by dialysis modality and cumulative incidence curves for death and transplantation. (A) Propensity-score matching of patients. (B) Cumulative incidence curves for death and transplantation, taking competing risks into account.

Cumulative survival probabilities from day 90 for PD and HD in the propensity score matched population were 99.3% vs. 98.9% (*P* = 0.633) at 6 months, 96.9% vs. 94.1% (*P* = 0.152) at 12 months, 95.8% vs. 90.6% (*P* = 0.044) at 18 months, and 94.3% vs. 87.6% (*P* = 0.022) at 24 months, respectively (Figure 1A). PD had a 51% lower risk of death than HD (HR 0.49, 95% CI 0.25–0.97; Table 3). The cause-specific survival was analyzed considering the competing risks events, death and transplantation, the difference was statistically significant for the death event (*P* = 0.040), but not for the transplantation event (*P* = 0.454) (Figure 1B).

**Table 3:** Hazard ratios of death for peritoneal dialysis compared with hemodialysis in propensity-score matched (n = 556) and all (n = 1,060) patients.

	Hazard ratio (95% confidence interval)	p-value
Propensity-score matched patients	0.49 (0.25–0.97)	0.040
All patients		
Unadjusted	0.57 (0.36–0.90)	0.015
Multivariable adjusted	0.63 (0.36–1.08)	0.095

**Figure 2:**
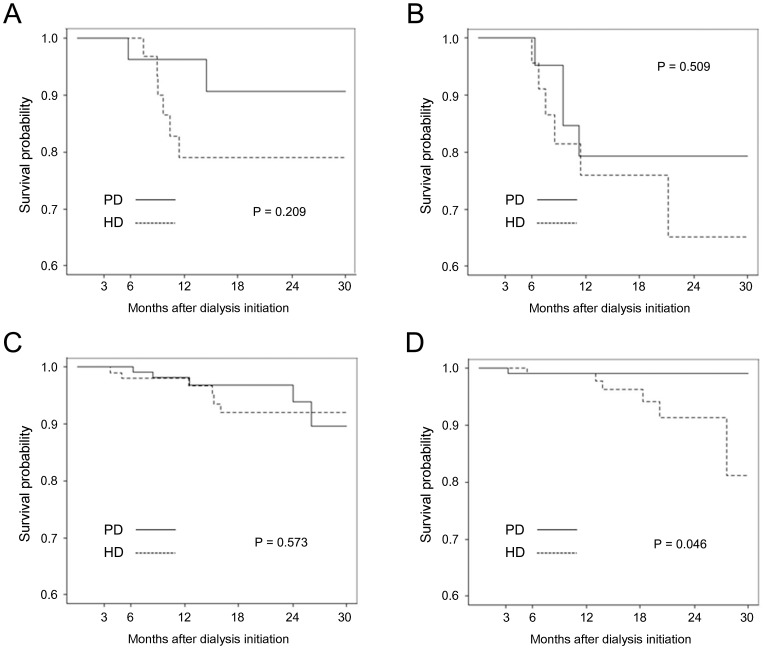
Kaplan-Meier curve according to age and presence of diabetes in propensity-score matched population. (A) age ≥65 years with diabetes; (B) ≥65 years without diabetes; (C) <65 years with diabetes; (D) <65 years without diabetes A ≥65 years, DM B ≥65 years, non-DM C <65 years, DM D <65 years, non-DM.

**Figure 3:**
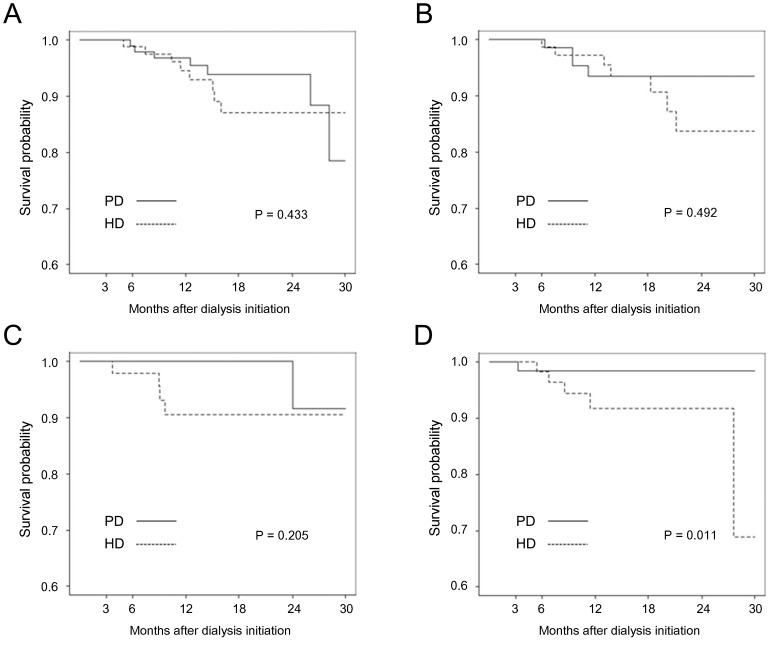
Kaplan-Meier curve according to sex and presence of diabetes in propensity-score matched population. (A) diabetic male; (B) non-diabetic male; (C) diabetic female; (D) non-diabetic female A Male, DM B Male, non-DM C Female, DM D Female, non-DM.

Kaplan-Meier survival curves for subgroups defined by age and the presence of diabetes mellitus are presented in Figure 2. The subgroup of patients aged less than 65 years without diabetes exhibited significantly better survival with PD than HD (*P* = 0.046), whereas the other three subgroups exhibited no significant differences in survival between the two modalities. Non-diabetic female patients on PD exhibited significantly superior survival than those on HD (*P* = 0.011), but diabetic female and male patients with or without diabetes exhibited no significant differences between the two dialysis modalities (Figure 3).

### Survival Analysis from Day 0 and Day 90 in All Patients

Cumulative survival probabilities from day 0 for PD and HD in all patients were 98.1% vs. 95.0% (*P* = 0.018) at 6 months, 95.0% vs. 91.3% (*P* = 0.034) at 12 months, 93.1% vs. 87.8% (*P* = 0.016) at 18 months, and 90.4% vs. 83.7% (*P* = 0.012) at 24 months, respectively (Figure S3A). The better survival of PD patients did not change when analyzing from day 90 (Figure S3B). The cause-specific survival from day 0 was analyzed considering the competing risks events, death and transplantation, the difference was statistically significant for the death event (*P* = 0.012), but not for the transplantation event (*P* = 0.387) (Figure S4A). Similar results were obtained in the analysis from day 90 as well (Figure S4B).

Multivariable Cox regression showed that PD tended to be associated with a 37% lower risk of death compared to HD (HR 0.63, 95% CI 0.36–1.08; Table 3). Older age, lower BMI, and fewer comorbidities, including congestive heart failure and peptic ulcer disease, was also significantly associated with increased mortality in the model (Table S3). Survival during the first 90 days was also analyzed for a supplementary analysis. During the first 90 days after initiating dialysis, PD patients exhibited a similar mortality risk as HD patients (HR 0.65, 95% CI 0.11–3.70).

## Discussion

In this study, we compared clinical outcomes between the two dialysis modalities in Korean ESRD patients. To the best of our knowledge, this study is the first nationwide prospective cohort study of the Asian ESRD population that compares outcomes between HD and PD. The influence of dialysis modality on patient survival is still somewhat controversial. Randomized controlled trials are ideal for obtaining this information, but a previous trial that compared clinical outcomes based on dialysis modality failed [12] due to the difficulty of randomizing against patient preferences. Alternatively, prospective cohort studies and skillful analyses with causal models have been attempted since the 2000s in order to overcome these limitations [9], [13]. However, only one prospective observational study in the Asian ESRD population has been performed: 83 PD and 83 HD patients were recruited in a regional area of Japan. This study reported similar mortality between the two dialysis modalities in Japanese prevalent ESRD patients [14].

We analyzed the clinical outcomes of ESRD patients using the propensity score matching method, which was used recently to control confounding factors [15], in order to overcome the limitation of non-random allocation to dialysis modality. In the Korean ESRD population, PD patients were younger and had a lower BMI than HD patients. The proportion of PD patients with diabetes as the primary renal disease or comorbid conditions, such as chronic lung disease and malignancy, was also lower compared to HD patients. Moreover, PD patients had higher hemoglobin levels and preserved higher 24 hr urine volumes than HD patients at the commencement of dialysis. These better baseline characteristics of PD patients were concordant with data from other countries, such as the United States and Canada [6], [9], [16], and resulted in younger and non-diabetic HD patients with lower BMI being selected for matching. Although HD patients were positively selected for matching, PD had a better survival rate in the early period of dialysis after balancing all of the measured baseline characteristics between the two dialysis modalities using propensity score matching. The superior outcomes of PD compared to HD in the early period of dialysis are in accordance with several previous studies from different cohorts.

A comparison of clinical outcomes between the two dialysis modalities found a survival advantage of PD relative to HD in the first 2 years among US Medicare patients treated with dialysis therapy [17], the Canadian dialysis population [18], Danish registry data [19], and US DaVita database [20].

On the other hand, the CHOICE study group reported that the adjusted risk of death did not differ between two dialysis modalities during the first year, but it was significantly higher among PD patients in the second year [6]. The Netherlands Cooperative Study on Adequacy of Dialysis (NECOSAD) study group also reported a similar mortality rate within 2 years of dialysis and better outcomes later for HD compared to PD [7].

In this cohort, the difference in cumulative survival probabilities between PD and HD seemed to increase over time despite it being the early period of dialysis with a mean follow-up of 16.3 months. This pattern is different from other registry data. The Australian and New Zealand Dialysis and Transplant (ANZDATA) Registry reported a crossover point of survival for PD vs. HD nearly 1 year after the initiation of dialysis [13]. The US retrospective cohort data analyzed by Weinhandl *et al.* also reported a crossover point of survival between the two modalities nearly 2 years in an analysis from day 90 [9]. A longer follow-up of our cohort population will provide information regarding whether the survival curve in Korean dialysis patients has a crossover point or progressively increasing difference in survival.

As propensity score matching was performed in the population surviving on day 90 in our cohort, the patients who died before day 90 were excluded from that step. In general, dialysis modality is highly subject to change in the early days, and we determined that day 90– by the time the individual modality is usually agreed upon – was a proper time-point from which survival could be evaluated, like in many other previous studies [9], [13], [21].

A supplementary analysis of data from the first 90 days revealed that 26% of deaths occurring in the first year of dialysis occurred within the first 90 days, which is similar to the 32% reported by Soucie *et al*. [22]. In addition, the two dialysis modalities exhibited similar mortality within the first 90 days. Subgroup analyses of the first 90 days in several cohorts revealed conflicting results. An elderly ESRD cohort from the US found that PD patients had a 16% higher rate of death during the first 90 days of renal replacement therapy than HD patients [23]. However, the ANZDATA registry reported lower mortality for PD compared to HD in the first 90 days; the reason was likely catheter use in HD patients who began renal replacement therapy [13]. Patients who start dialysis urgently are treated almost exclusively with HD and frequently use tunneled or non-tunneled catheters. The use of a catheter in HD is associated with a higher mortality rate [24], [25]. However, no difference in survival between PD and HD was previously reported in an analysis of patients who started dialysis electively [16]. In contrast to the relatively lower rates of catheter use in Australia and New Zealand compared to international standards [24], [26], Korean dialysis patients who were initially assigned to HD had higher rates of catheter use [27]. In this cohort, 436 (59.2%) HD patients used a catheter for HD at day 90. Despite these higher rates of catheter use in HD patients, our cohort did not confirm better survival in PD relative to HD within 90 days initiating dialysis.

In subgroup analyses of this study, PD patients without diabetes younger than 65 years old showed superior outcome than HD patients as many previous studies shown advantage of PD in non-diabetic younger patients [6], [8], [10], [28]. Sex differences and the presence of diabetes could affect survival in ESRD patients [8], [17], [21], [29]. The NECOSAD study reported that women with diabetes have a higher mortality risk with PD or HD compared to their male counterparts [29]. The European Renal Association-European Dialysis and Transplant Association (ERA-EDTA) Registry reported that diabetic females tend to have increased mortality risk with PD, whereas PD had survival benefits, independent of diabetes, in males [21]. Previous studies have suggested that lower BMI and glucose load are contributing factors that increase the risk of death in female PD patients. In accordance with a previous study [29], subgroup analysis according to sex and diabetes revealed significantly superior survival among PD patients compared to HD patient in only the non-diabetic female group.

The US Renal Data System (USRDS) and ANZDATA Registry reported that the most common cause of death is cardiovascular disease, which was attributed to more than twice as many deaths as infectious disease, the second leading cause of death in ESRD patients [30], [31], [32]. Infectious complication has rarely been reported as a common cause of death in dialysis patients. Only one report stated that cardiac and infectious diseases were the main causes of death; they were the same percentages in a dialysis population in Israel in the 1990s [33]. The Israeli population has a small percentage of diabetes as the primary renal disease, whereas more than 40% of our prospective cohort had diabetes. Causable comorbidities, such as congestive heart failure, coronary artery disease, and peripheral vascular disease, in our cohort were much lower than the ANZDATA registry [32] or US population [30], which can be one of the reasons why cardiovascular complications are a relatively less common cause of death than infectious complications. In addition, a relatively higher use of catheter (>50%) in patients who started HD may affect the higher infectious mortality in our cohort.

Our study has several potential limitations. The dialysis modalities were not randomly assigned and the study subjects were followed up for a relatively short duration. In addition, we could not avoid selection bias, which means the some of the too ill or too healthy dialysis patients were not included. Nevertheless, this study is the first prospective Asian cohort study comparing mortality outcome based on dialysis modality in nationwide multi-centers, including primary dialysis centers and tertiary university hospitals.

In conclusion, Korean patients undergoing incident dialysis have distinct characteristics; PD patients were younger and had fewer comorbidities than HD patients. After adjusting for these factors using propensity scores, PD still had superior outcomes than HD in the early period of dialysis. Notably, the most common cause of death was infection in this cohort. Further analysis of data with longer follow-up is needed to suggest a survival benefit of a particular dialysis modality.

## Supporting Information

Figure S1 **Consort diagram for patient selection.**
(TIF)

Figure S2 **Distribution of propensity score before and after matching.**
(TIF)

Figure S3 **Survival probability by dialysis modality in all patients (unadjusted).** The survival probability from day 0 (A) and day 90 (B) by dialaysis modality.
(TIF)

Figure S4 **Cumulative incidence curves for death and transplantation.** Evaluation of the competing risks in all patients from day 0 (A) and day 90 (B).
(TIF)

Table S1 **Comparison of patient characteristics between propensity-score matched and unmatched population.**
(DOCX)

Table S2 **Deaths as period from initiation of dialysis (n = 1,060).**
(DOCX)

Table S3 **Hazard ratios for mortality with other covariates included in the multivariable Cox regression.**
(DOCX)
