# Supplementary figures and images for: Survival Advantage of Peritoneal Dialysis Relative to Hemodialysis in the Early Period of Incident Dialysis Patients: A Nationwide Prospective Propensity-Matched Study in Korea
Source: PLoS One. 2013 Dec 30;8(12):e84257. doi: 10.1371/journal.pone.0084257 (PMC3875495; doi:10.1371/journal.pone.0084257)

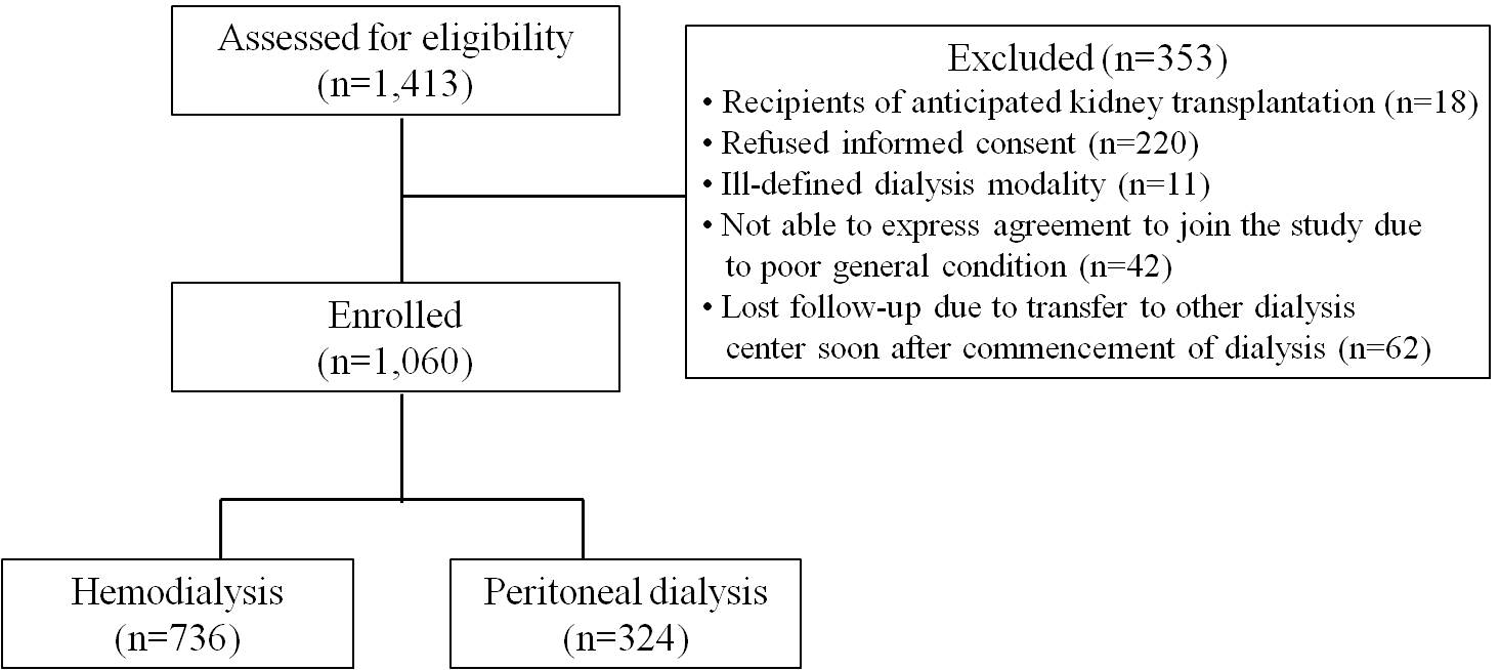

Supplement: Figure S1 — Consort diagram for patient selection. (TIF) [file pone.0084257.s001.tif]

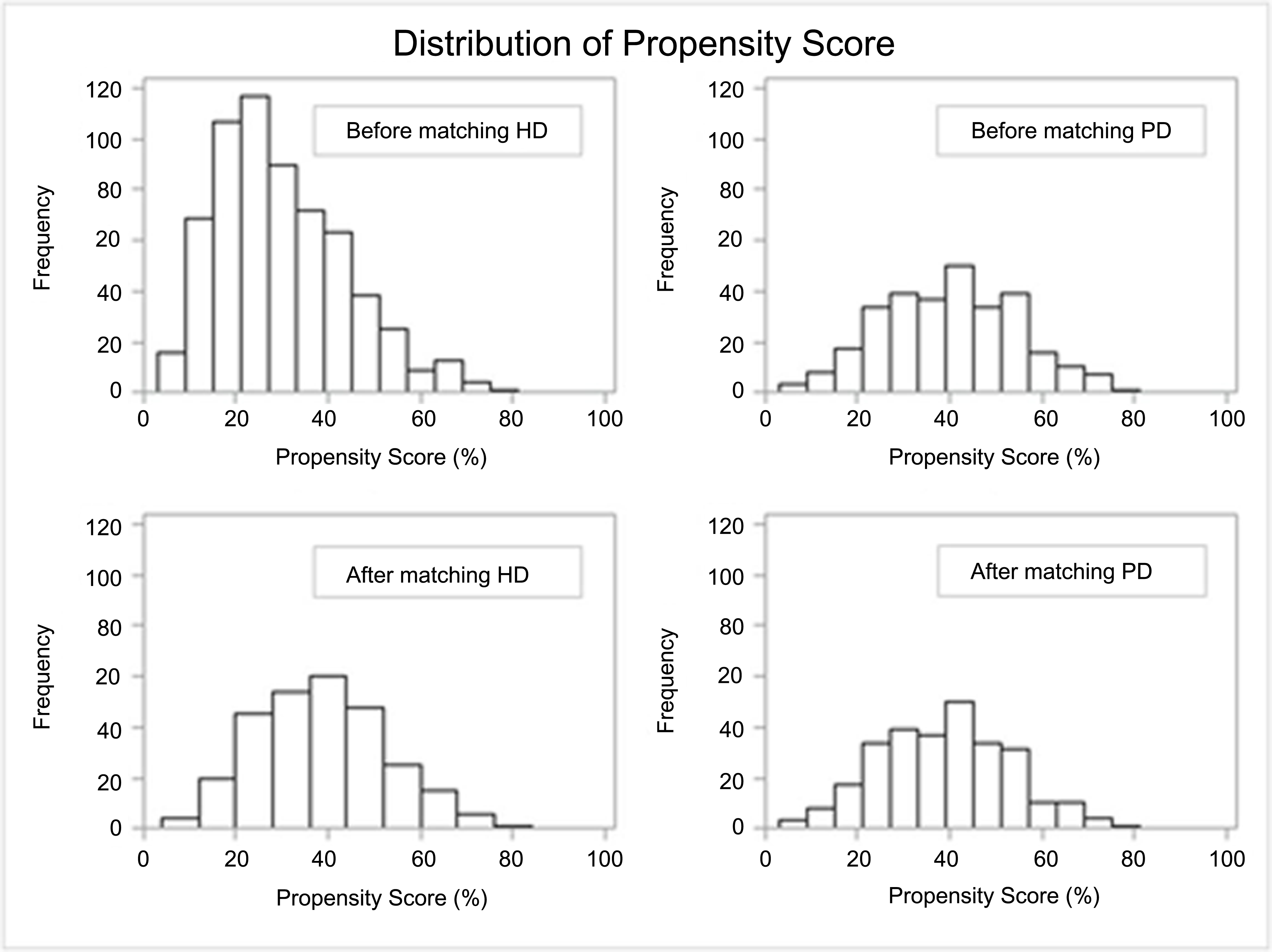

Supplement: Figure S2 — Distribution of propensity score before and after matching. (TIF) [file pone.0084257.s002.tif]

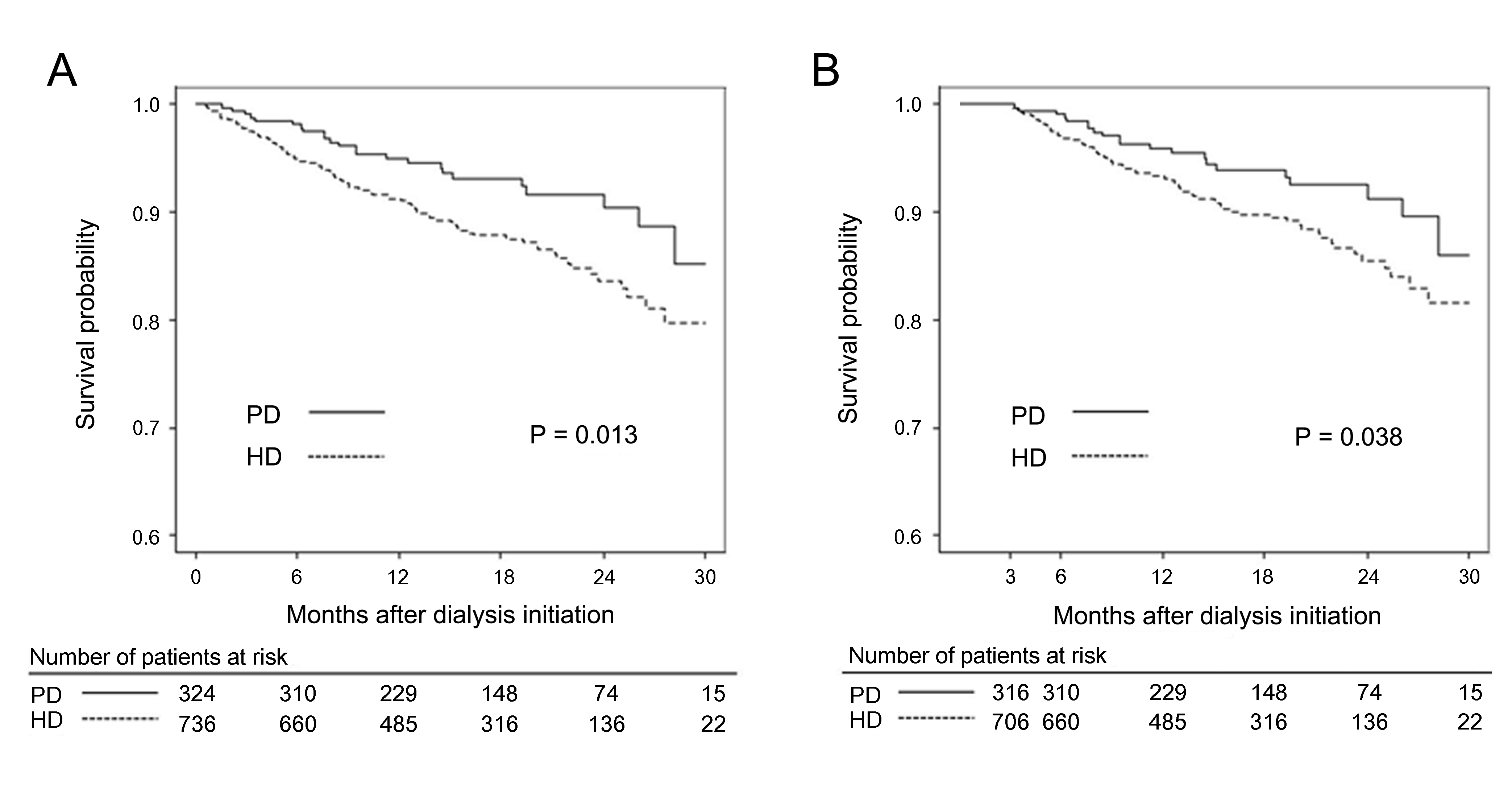

Supplement: Figure S3 — Survival probability by dialysis modality in all patients (unadjusted). The survival probability from day 0 (A) and day 90 (B) by dialaysis modality. (TIF) [file pone.0084257.s003.tif]

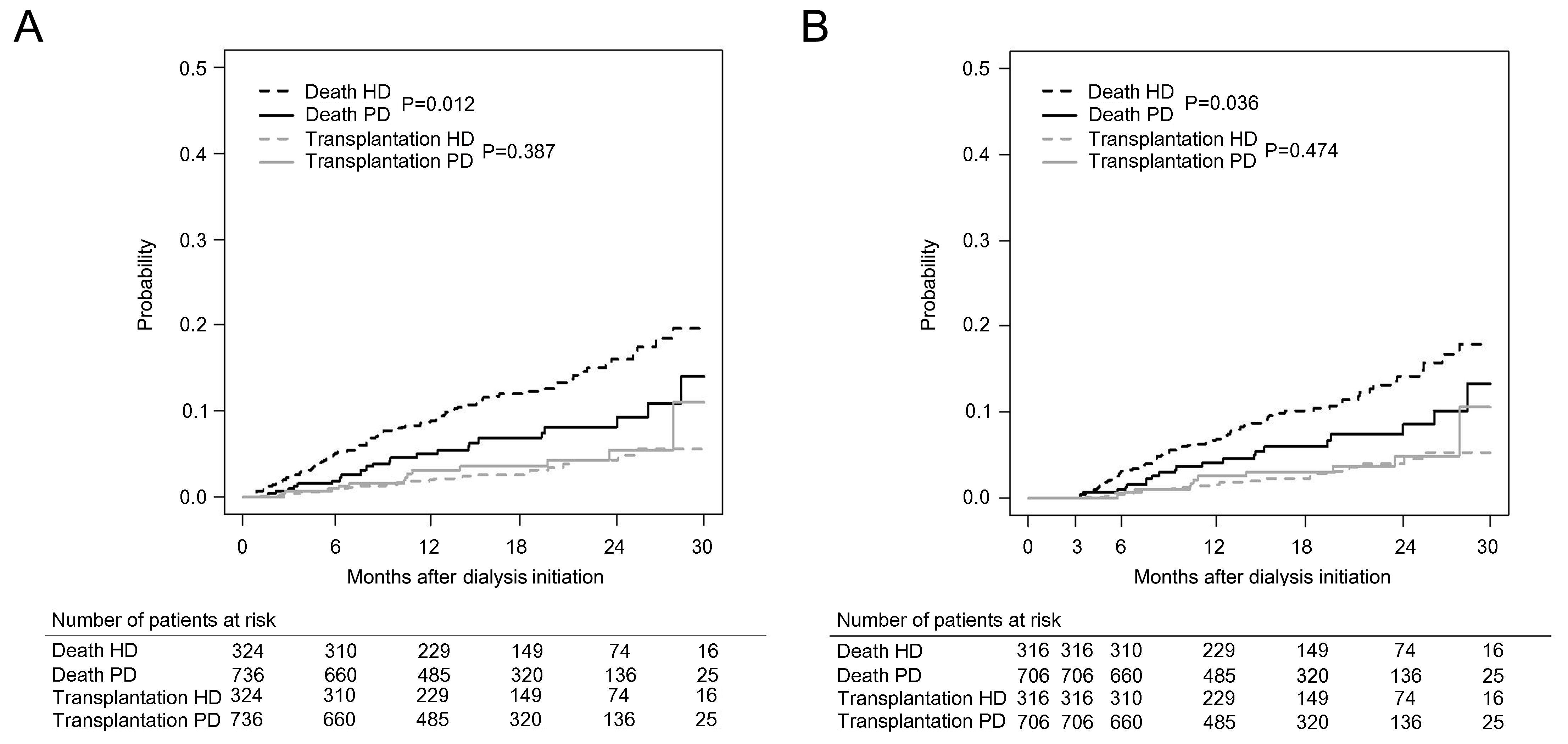

Supplement: Figure S4 — Cumulative incidence curves for death and transplantation. Evaluation of the competing risks in all patients from day 0 (A) and day 90 (B). (TIF) [file pone.0084257.s004.tif]
