# Supplementary material for: Survival Advantage of Peritoneal Dialysis Relative to Hemodialysis in the Early Period of Incident Dialysis Patients: A Nationwide Prospective Propensity-Matched Study in Korea
Source: PLoS One. 2013 Dec 30;8(12):e84257. doi: 10.1371/journal.pone.0084257 (PMC3875495; doi:10.1371/journal.pone.0084257)
Supplement: Table S1 — Comparison of patient characteristics between propensity-score matched and unmatched population. (DOCX) [file pone.0084257.s005.docx]

Table S1. Comparison of patient characteristics between propensity-score matched and unmatched population.

| Characteristics |  | Patients who survived until day 90 (n=1,022) | | | | | | | |
| --- | --- | --- | --- | --- | --- | --- | --- | --- | --- |
|  |  | Matched population (n=556) | | |  | Unmatched population (n=466) | | | |
|  |  | Matched HD  (n=278) | Matched PD  (n=278) | P value |  | Unmatched HD  (n=428) | P value^†^ | Unmatched PD  (n=38) | P value^†^ |
| Age at initiation of dialysis (years) |  | 51.9±14.5 | 51.6±13.0 | 0.770 |  | 61.6±12.2 | 0.000 | 48.0±15.4 | 0.123 |
| Sex (Male%) |  | 169 (60.8) | 168 (60.4) | 1.000 |  | 258 (60.3) | 0.937 | 22 (57.9) | 0.860 |
| Body mass index (kg/m^2^) |  | 22.7±3.0 | 22.7±3.3 | 1.000 |  | 23.7 ±3.7 | 0.000 | 22.3±3.3 | 0.446 |
| Primary renal disease, n (%) | | | | | | | | | |
| Diabetes |  | 130 (50.8) | 126 (47.7) | 0.282 |  | 223 (60.8) | 0.024 | 15 (46.9) | 1.000 |
| Hypertension |  | 47 (18.4) | 53 (20.1) |  |  | 67 (18.3) |  | 6 (18.8) |  |
| Glomerulonephritis |  | 47 (18.4) | 62 (23.5) |  |  | 40 (10.9) |  | 8 (25.0) |  |
| Others |  | 32 (12.5) | 23 (8.7) |  |  | 37 (10.1) |  | 3 (9.4) |  |
| Comorbidity at initiation of dialysis | | | | | | | | | |
| Congestive heart failure |  | 37 (13.3) | 36 (12.9) | 1.000 |  | 60 (15.0) | 0.578 | 5 (15.2) | 0.785 |
| Coronary artery disease |  | 27 (9.7) | 28 (10.1) | 1.000 |  | 71 (17.8) | 0.004 | 4 (12.1) | 0.760 |
| Peripheral vascular disease |  | 16 (5.8) | 15 (5.4) | 1.000 |  | 48 (12.0) | 0.007 | 2 (6.7) | 0.676 |
| Arrhythmia |  | 3 (1.1) | 4 (1.4) | 1.000 |  | 14 (3.5) | 0.077 | 1 (3.0) | 0.431 |
| Cerebrovascular disease |  | 16 (5.8) | 20 (7.2) | 0.606 |  | 71 (17.8) | 0.000 | 8 (25.0) | 0.004 |
| Chronic lung disease |  | 17 (6.1) | 15 (5.4) | 0.856 |  | 68 (12.5) | 0.000 | 1 (3.1) | 1.000 |
| Peptic ulcer disease |  | 16 (5.8) | 21 (7.6) | 0.497 |  | 38 (9.5) | 0.084 | 0 (0.0) | 0.145 |
| Moderate to severe chronic liver disease |  | 10 (3.6) | 10 (3.6) | 1.000 |  | 6 (1.5) | 0.120 | 0 (0.0) | 0.607 |
| Connective tissue disease |  | 24 (8.6) | 29 (10.4) | 0.564 |  | 43 (10.8) | 0.433 | 3 (9.1) | 1.000 |
| Malignancy |  | 14 (5.0) | 8 (2.9) | 0.277 |  | 42 (10.6) | 0.011 | 0 (0.0) | 1.000 |
| Laboratory data at initiation of dialysis | | | | | | | | | |
| Hemoglobin (g/dL) |  | 9.2±1.7 | 9.1±1.6 | 0.516 |  | 8.6±1.7 | 0.000 | 9.6±1.7 | 0.061 |
| Blood urea nitrogen (mg/dL) |  | 78.0±32.1 | 79.2±36.0 | 0.680 |  | 81.5±40.4 | 0.200 | 72.2±35.4 | 0.259 |
| Creatinine (mg/dL) |  | 8.4±3.3 | 8.6±3.7 | 0.717 |  | 8.1±3.7 | 0.202 | 8.4±2.7 | 0.775 |
| Albumin (g/dL) |  | 3.5±0.7 | 3.4±0.7 | 0.324 |  | 3.3±0.7 | 0.000 | 3.3±0.8 | 0.618 |
| Ca (mg/dL) |  | 7.8±1.1 | 7.7±1.1 | 0.790 |  | 7.7±1.0 | 0.842 | 7.8±1.3 | 0.880 |
| P (mg/dL) |  | 5.5±1.7 | 5.5±1.8 | 0.705 |  | 5.4±2.0 | 0.662 | 5.1±1.9 | 0.169 |
| Estimated GFR (ml/min/1.73m^2^) |  | 7.1±3.2 | 7.2±3.6 | 0.630 |  | 7.7±4.8 | 0.042 | 7.2±3.0 | 0.998 |
| Urine volume (ml/day) |  | 782.6±673.7 | 763.7±664.8 | 0.740 |  | 537.9±556.7 | 0.000 | 778.5±683.9 | 0.898 |

^†^Matched vs. unmatched population

Data are expressed as number (%) or mean±standard deviation.

P-values were estimated by chi-square, Fisher’s exact and student *t* tests as appropriate. Abbreviations: HD, hemodialysis; PD, peritoneal dialysis; GFR, glomerular filtration rate.
