# Supplementary material for: Survival Advantage of Peritoneal Dialysis Relative to Hemodialysis in the Early Period of Incident Dialysis Patients: A Nationwide Prospective Propensity-Matched Study in Korea
Source: PLoS One. 2013 Dec 30;8(12):e84257. doi: 10.1371/journal.pone.0084257 (PMC3875495; doi:10.1371/journal.pone.0084257)
Supplement: Table S2 — Deaths as period from initiation of dialysis (n = 1,060). (DOCX) [file pone.0084257.s006.docx]

Table S2. Deaths as period from initiation of dialysis (n=1,060).

|  | HD | PD | Total |
| --- | --- | --- | --- |
| Deaths within 90 days after initiation of dialysis | 16/89 (18.0%) | 3/24 (12.5%) | 19/113 (16.8%) |
| Deaths within 1 year after initiation of dialysis | 58/89 (65.2%) | 15/24 (62.5%) | 73/113 (64.6%) |
| Death within 90 days / Death within 1 year (%) | 16/58 (27.6%) | 3/15 (20.0%) | 19/73 (26.0%) |
