# Supplementary material for: Survival Advantage of Peritoneal Dialysis Relative to Hemodialysis in the Early Period of Incident Dialysis Patients: A Nationwide Prospective Propensity-Matched Study in Korea
Source: PLoS One. 2013 Dec 30;8(12):e84257. doi: 10.1371/journal.pone.0084257 (PMC3875495; doi:10.1371/journal.pone.0084257)
Supplement: Table S3 — Hazard ratios for mortality with other covariates included in the multivariable Cox regression. (DOCX) [file pone.0084257.s007.docx]

Table S3. Hazard ratios for mortality with other covariates included in the multivariable Cox regression.

|  | Hazard Ratio | 95% CI | P value |
| --- | --- | --- | --- |
| Age (years) | 1.05 | 1.03-1.07 | < 0.001 |
| Male | 1.38 | 0.82-2.34 | 0.231 |
| Body mass index (kg/m^2^) | 0.91 | 0.85-0.98 | 0.014 |
| Diabetes as primary renal disease | 1.04 | 0.45-2.41 | 0.926 |
| Hemoglobin (g/dL) | 1.00 | 0.94-1.06 | 0.865 |
| Blood urea nitrogen (per 10 mg/dL) | 1.00 | 0.99-1.00 | 0.321 |
| Crearinine (mg/dL) | 1.03 | 0.97-1.08 | 0.327 |
| Serum albumin (g/dL) | 0.76 | 0.51-1.12 | 0.166 |
| Ca (mg/dL) | 1.00 | 1.00-1.02 | 0.196 |
| P (mg/dL) | 1.00 | 0.94-1.06 | 0.971 |
| 24hr Urine volume (per 100 ml/day) | 1.00 | 0.99-1.00 | 0.183 |
| Congestive heart failure | 2.28 | 1.31-3.96 | 0.004 |
| Coronary artery disease | 1.10 | 0.61-1.97 | 0.754 |
| Peripheral vascular disease | 1.51 | 0.81-2.78 | 0.192 |
| Arrhythmia | 1.96 | 0.66-5.78 | 0.223 |
| Cerebrovascular disease | 0.80 | 0.39-1.63 | 0.541 |
| Chronic lung disease | 1.42 | 0.79-2.52 | 0.239 |
| Peptic ulcer disease | 0.12 | 0.03-0.52 | 0.005 |
| Moderate to severe chronic liver disease | 2.20 | 0.83-5.83 | 0.115 |
| Connective tissue disease | 1.61 | 0.82-3.18 | 0.168 |
| Malignancy | 1.74 | 0.82-3.67 | 0.148 |

CI, confidence interval; HD, hemodialysis; PD, peritoneal dialysis; BMI, body mass index
